# Exploring the role of gut microbiota in Parkinson’s disease: insights from fecal microbiota transplantation

**DOI:** 10.3389/fnins.2025.1574512

**Published:** 2025-06-13

**Authors:** Mengqi Guo, Han Gao, Yuan Wang, Yuanyuan Xiang

**Affiliations:** ^1^Department of Neurology, Shandong Provincial Hospital, Affiliated to Shandong First Medical University, Jinan, Shandong, China; ^2^Department of Neurology, Shandong First Medical University, Jinan, Shandong, China; ^3^Department of Ultrasound, Shandong Provincial Hospital, Affiliated to Shandong First Medical University, Jinan, Shandong, China; ^4^Department of Ultrasound, Shandong First Medical University, Jinan, Shandong, China

**Keywords:** Parkinson’s disease, gut microbiota, gut-brain axis, fecal microbiota transplantation, gut dysbiosis

## Abstract

As a common neurodegenerative disease, Parkinson’s disease (PD) is typified by *α*-synuclein (*α*-syn) aggregation and progressive degeneration of dopaminergic neurons within the substantia nigra. Clinical manifestations encompass motor symptoms and non-motor aspects that severely impair quality of life. Existing treatments mainly address symptoms, with no effective disease-modifying therapies available. The gut microbiota refers to the community of microorganisms that colonize the intestinal tract. The gut microbiota, gut, and brain are all connected via a complicated, mutual communication pathway known as the “gut microbiota-gut-brain axis.” Gut microbiota dysbiosis is strongly linked to the onset and course of PD, according to growing data. In individuals with PD, gut dysbiosis correlates with clinical phenotype, disease duration, severity, and progression rates. Mechanistically, gut dysbiosis contributes to PD through enhanced intestinal permeability, increased intestinal inflammation and neuroinflammation, abnormal *α*-syn aggregation, oxidative stress, and reduced neurotransmitter synthesis. Therefore, focusing on the gut microbiota is regarded as a potentially effective treatment strategy. Fecal microbiota transplantation (FMT) is an emerging approach to modulate gut microbiota, with the goal of recovering microbiota diversity and function by transferring functional intestinal flora from healthy individuals into patients’ gastrointestinal tracts. FMT is expected to become a promising therapy of PD and has a broad research and application prospect. Evidence suggests that FMT may restore gut microbiota, ease clinical symptoms, and provide potential neuroprotective benefits. However, the precise therapeutic mechanisms of FMT in PD remain uncertain, necessitating further research to clarify its effectiveness. This review examines alterations in gut microbiota linked to PD, mechanisms through which gut dysbiosis influences the disease, and the latest advancements in FMT research for treating PD, setting the stage for its clinical application.

## 1 Introduction

As a prevalent neurodegenerative illness, Parkinson’s disease (PD) is typified by aberrant *α*-synuclein (*α*-syn) folding and aggregation, as well as dopaminergic neurons degenerating and losing within the substantia nigra (SN; Lajoie et al., 2021). It has a complicated and multifaceted etiology that includes environmental, genetic, and other variables. Its pathogenesis mainly involves *α*-syn abnormal aggregation (Tofaris, 2022), neuroinflammation (Marogianni et al., 2020), oxidative stress (Ding et al., 2018), and mitochondrial dysfunction (Malpartida et al., 2021).

According to statistics, in 2015, the number of PD cases worldwide was approximately 6.2 million, and by 2040, that figure is predicted to reach 12.9 million (Dorsey and Bloem, 2018). The incidence of PD is significantly related to age (Qi et al., 2021). The typical course is insidious, slow, and progressive. Clinical manifestations encompass motor aspects like bradykinesia, resting tremor, and rigidity, along with non-motor aspects like gastrointestinal dysfunction and sleep disturbances, which often more significantly affect the quality of life. According to statistical data, from 1999 to 2019, the death rate for PD rose from 5.4 per 100,000 to 8.8 per 100,000 (Rong et al., 2021). The growth rate of PD-related disabilities and deaths is faster than that of any other neurological disease (The Lancet, 2022).

Currently, the treatment of PD is mainly symptomatic, comprising pharmacotherapy and surgical interventions. However, effective disease-modifying therapies are still lacking. Commonly utilized medications in clinical treatment include the dopamine (DA) precursor levodopa, catechol-O-methyltransferase inhibitors, monoamine oxidase B inhibitors, anticholinergic drugs, and DA receptor agonists. Among these, DA replacement therapy remains the first-line intervention for PD, but it only partially improves motor symptoms, without alleviating non-motor symptoms or delaying disease progression.

Furthermore, its efficacy tends to decline after 3–5 years, and prolonged use may produce adverse effects such as the wearing-off phenomenon, the on–off phenomenon, and dyskinesia, resulting in poor patient tolerance (Armstrong and Okun, 2020). Deep brain stimulation, the main surgical treatment for PD, alleviates symptoms by implanting electrodes into specific brain nuclei and delivering microcurrents to stimulate target areas, thereby modifying electrical signals in related neural circuits (Sharma et al., 2020). However, deep brain stimulation controls symptoms without curing the disease. Patients still require long-term medication post-surgery, and the associated medical costs remain high. Currently, no existing therapy has shown significant efficacy in reversing *α*-syn aggregation, restoring DA neuron degeneration, or delaying disease progression (Suarez-Cedeno et al., 2017).

Recent research has introduced a new perspective: the central nervous system (CNS) may be impacted by the gut microbiota. Gut microbiota is known as the collection of bacteria, viruses, fungi, and other microorganisms inhabiting the intestinal tract, representing the largest and most intricate microflora in the human body. It includes approximately 50 bacterial phyla. Bacteroidetes and Firmicutes comprise more than 90% of the total (Ding et al., 2021).

Under normal circumstances, the relative abundance and diversity of gut microbiota are dynamically balanced, influenced by variables including diet, stress and antibiotics (Sun et al., 2022). Many physiological processes, including nutrient digestion and absorption, energy metabolism, immune function regulation, neural function modulation, and intestinal barrier maintenance, are influenced by the gut microbiota. It is necessary for the immune, endocrine, and nervous systems to grow and mature. Dysbiosis of gut microbiota refers to disturbances in overall microbial composition and the relative abundance of specific flora, disrupting the body’s homeostasis. In preclinical studies, gut dysbiosis has been linked to the pathophysiological mechanisms of intestinal diseases (e.g., inflammatory bowel disease, irritable bowel syndrome), mental disorders (e.g., anxiety, depression, autism spectrum disorder), as well as neurological diseases (e.g., multiple sclerosis, Alzheimer’s disease [AD], PD, amyotrophic lateral sclerosis; Mou et al., 2022; Sorboni et al., 2022; Kujawa et al., 2023; Solanki et al., 2023; Loh et al., 2024).

Modifications to gut microbiota abundance or diversity and its metabolites have been observed in animal models and clinical cases of PD, highlighting the significance in the onset and progression of the disease. Addressing gut dysbiosis offers PD patients a potential treatment strategy. This article reviews the characteristic changes in gut microbiota among PD patients, the potential mechanisms by which gut dysbiosis may contribute to PD pathogenesis, and recent advancements in fecal microbiota transplantation (FMT) for PD treatment, providing a scientific foundation for future clinical applications.

## 2 Gut microbiota and the connection with PD

### 2.1 Gut microbiota-gut-brain axis

Recent research has revealed a sophisticated bidirectional communication pathway connecting the gut microbiota, the gut, and the brain, termed the “gut microbiota-gut-brain axis” (MGBA; Cryan and Dinan, 2012). This axis enables crosstalk between the enteric nervous system (ENS) and the CNS by means of neurological, immunological, endocrine, and metabolic signaling pathways (Matheson and Holsinger, 2023). Dysregulation of this axis has been associated with the pathophysiology of PD.

Braak et al. (2006) first proposed the hypothesis that the origins of PD may be in the gastrointestinal tract. They proposed that a neurotropic pathogen crossing the gastric epithelium could induce *α*-syn misfolding in the ENS, then propagate to the brain via retrograde axonal transport through a chain of interconnected neurons, driving PD pathology. This hypothesis aligns with clinical observations that gastrointestinal symptoms frequently appear before motor problems in PD patients. According to epidemiological research, patients with inflammatory bowel disease are more likely to acquire PD than people without inflammatory bowel disease (Brudek and van Laar, 2019). Furthermore, vagotomy performed to treat peptic ulcers has been shown to reduce PD risk (Sun et al., 2022). These findings underscore the strong association between PD and the gastrointestinal tract, indirectly supporting the hypothesis of intestinal origin.

Preclinical studies provide additional evidence. Kim et al. (2019) found that *α*-syn was transferred from the gut, initially appearing in the vagus nerve’s dorsal motor nucleus nerve and eventually reaching the SN compacta via sequential diffusion and transmission. Importantly, vagotomy effectively prevented *α*-syn transfer from the colon to the brain, confirming the role of the vagus nerve. Similarly, Bhattarai et al. (2021) administered rotenone to both germ-free and conventionally raised mice for 6 weeks. Although tyrosine hydroxylase neurons were lost in each group, only conventionally raised mice exhibited decreased motor strength and coordination, emphasizing the importance of gut microbiota in PD etiology. Collectively, these findings highlight the critical function of this axis in the onset and course of PD.

### 2.2 Dysbiosis of gut microbiota in PD patients

Numerous case–control investigations have researched the gut microbiota composition of PD patients. While findings vary due to differences in sample size, inclusion and exclusion criteria, experimental design, and individual factors (e.g., age, diet, geography, and genetic background; Li Z. et al., 2022), certain consistent trends have emerged. For example, compared with healthy controls, PD patients exhibit higher abundances of certain genera, including *Bilophila*, *Akkermansia*, Verrucomicrobia, *Lactobacillus*, and *Parabacteroides*, alongside lower abundances of beneficial bacteria, especially Lachnospiraceae, *Roseburia*, *Faecalibacterium*, *Blautia*, and *Prevotella* (Scheperjans et al., 2014; Li et al., 2017; Barichella et al., 2018; Lin et al., 2019; Lubomski et al., 2019; Cirstea et al., 2020; Nishiwaki et al., 2020a; Yu et al., 2023). Furthermore, specific microbial taxa correlate with clinical phenotypes, disease duration, severity, and progression rate. Table 1 summarizes these associations, illustrating the complicated link between microbiota changes and PD pathology.

**Table 1:** Association between gut microbiota and PD phenotypes.

Ref.	Microbiota changes	Aspects of impact	Correlation
Scheperjans et al. (2014)	Enterobacteriaceae↑	disease severity (postural instability and gait disturbances)	positive
Li et al. (2017)	*Enterococcus*, *Proteus*, *Escherichia*-Shigella↑	disease severity and PD duration	positive
	*Blautia*, *Faecalibacterium*, *Ruminococcus*↓		negative
Barichella et al. (2018)	Lachnospiraceae↓	disease severity (postural instability, gait disturbances and cognitive impairment)	positive
	Lactobacillaceae↑, Christensenellaceae↑		
Lin et al. (2019)	*Bacteroides*	motor symptom	positive
		in PD patients: with tremor subtype<with non-tremor subtype	---
Aho et al. (2021)	microbial alpha diversity indices	disease severity	positive
Mao et al. (2021)	*Klebsiella*, *Parasutterella*	disease severity and PD duration	positive
	hydrogen-generating *Prevotella*	disease severity	negative
Zheng et al. (2021)	*Lactobacillus gasseri*, Deferribacterales	disease duration	positive
	*Escherichia*/*Shigella*, Lachnospiraceae, *Clostridium coccoides*		negative
	Enterobacteriaceae, *Proteus*, *Escherichia*, *Enterococcus*, Lactobacillaceae	disease severity	positive
	Lachnospiraceae, *Blautia*, *Ruminococcus*, *Faecalibacterium*		negative
Cilia et al. (2021)	*Roseburia* (Firmicutes phylum) at baseline↓	disease severity	positive
	Ruminococcaceae and Actinobacteria at baseline↓	faster cognitive impairment	
Nishiwaki et al. (2022)	SCFA-producing genera, *Blautia*, *Fusicatenibacter*, *Faecalibacterium*↓	accelerated disease progression	positive
	mucin-degrading genus *Akkermansia*↑		

### 2.3 Potential mechanisms of gut microbiota dysbiosis in PD pathogenesis

**Figure 1:**
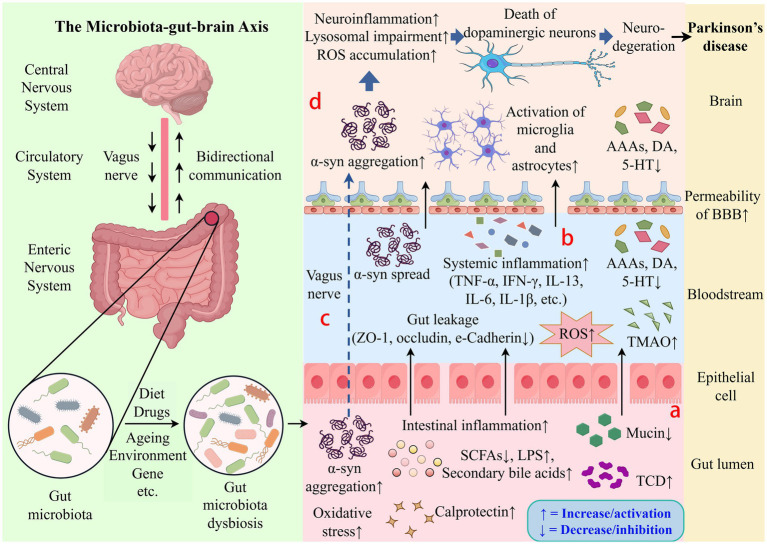
Schematic diagram of potential mechanisms by which gut microbiota influences Parkinson’s disease. The gut microbiota-gut-brain axis can affect the onset and course of PD. **(a)** Dysbiosis is associated with increased intestinal permeability, dysregulated gut microbiota metabolites, exacerbated oxidative stress and intestinal inflammation. **(b)** Gut leakage can trigger inflammatory cytokines to be released, resulting in systemic inflammation. **(c)** The vagus nerve allows abnormal α-syn aggregation to spread from the ENS to the CNS. **(d)** Pro-inflammatory cytokines can penetrate the blood–brain barrier, cause the activation of microglia and astrocytes, and aggravate neuroinflammation, which results in dopaminergic neuron loss and degeneration. ROS, Reactive Oxygen Species; AAAs, Aromatic Amino Acids; DA, Dopamine; 5-HT, 5-Hydroxytryptamine; *α*-syn, *α*-synuclein; BBB, Blood–Brain Barrier; TNF-*α*, Tumor Necrosis Factor-alpha; IFN-*γ*, Interferon-gamma; IL-13, Interleukin-13; IL-6, Interleukin-6; IL-1β, Interleukin-1 beta; ZO-1, Zonula Occludens-1; TMAO, Trimethylamine N-Oxide; SCFAs, Short-Chain Fatty Acids; LPS, Lipopolysaccharide; TCD, Total Cumulative Dose.

In recent years, research has increasingly supported the hypothesis that gut microbiota dysbiosis acts as a triggering factor for PD (Costa et al., 2022). Dysbiosis and its metabolites are thought to affect the onset and progression via several interconnected mechanisms, including raised intestinal permeability, exacerbated intestinal inflammation and neuroinflammation, aberrant *α*-syn aggregation, elevated oxidative stress, and reduced neurotransmitter manufacture (Figure 1).

#### 2.3.1 Increase in intestinal permeability

Dysbiosis of gut microbiota can compromise the intestinal epithelial barrier, leading to increased penetration. This “leaky gut” condition permits neuroactive small molecules, including potentially toxic metabolites derived from bacteria and microbial sources, to translocate into systemic circulation, accelerating pathological processes and elevating PD risk. Mucin, a key structural component of gastrointestinal mucosa, is essential for preserving barrier integrity. In PD patients, *Prevotella* deficiency is correlated to impaired mucin production, increased gut permeability, and disease progression (Scheperjans et al., 2014). Fang et al. (2024) demonstrated that chronic rotenone administration significantly reduced colonic mucus thickness and downregulated the expression of tight junction proteins (e.g., Zonula Occludens-1, occludin), confirming the essential role of gut microbiota in maintaining intestinal barrier integrity. Notably, FMT effectively alleviated rotenone-induced intestinal barrier impairment. The study further revealed that gut microbiota dysbiosis promotes excessive hydrogen sulfide production by sulfate-reducing bacteria, which subsequently degrades the mucus layer, disrupts the intestinal epithelial barrier, enhances intestinal permeability, and ultimately contribute to gut leakage (Munteanu et al., 2024). Short-chain fatty acids (SCFAs) contribute to intestinal barrier maintenance. According to studies, PD patients have much less bacteria that produce SCFA, resulting in lower fecal SCFA levels (Bisaglia, 2022). Experimental evidence suggests that administering butyrate to PD animal models can delay disease progression by improving motor function, preserving intestinal barrier integrity, reducing intestinal leakage, and secondary translocation of intestinal contents (Zheng et al., 2021).

#### 2.3.2 Aggravation of intestinal inflammation and neuroinflammation

According to compelling data, chronic intestinal inflammation and neuroinflammation are exacerbated by pro-inflammatory dysbiosis of the gut microbiota, which are thought to potentially contribute to the PD pathophysiology. Lin et al. (2019) reported elevated concentrations of pro-inflammatory cytokines, including Tumor Necrosis Factor-alpha (TNF-*α*), Interferon-gamma, and Interleukin-13, in the plasma of PD patients. There were positive correlations between the levels of TNF-α and Interferon-gamma and the abundance of *Bacteroides* and Verrucomicrobia, respectively. Additionally, fecal calprotectin, a hallmark of intestinal inflammation, was considerably increased in PD patients (Weis et al., 2019). Keshavarzian et al. (2015) used high-throughput ribosomal RNA sequencing to reveal that PD patients had lower “anti-inflammatory” bacteria, including *Blautia*, *Coprococcus*, and *Roseburia*, alongside more “pro-inflammatory” bacteria like *Faecalibacterium*. Preclinical studies demonstrate that dysbiosis exacerbates neuroinflammation through pathways such as Toll-like receptor 4 (TLR4)/Nuclear factor kappa-light-chain-enhancer of activated B cells (NF-κB), which raises the production of inflammatory markers, such as Glycogen synthase kinase 3 beta, inducible nitric oxide synthase, and Interleukin-1 beta, along with activating microglia and astrocytes in the SN (Varesi et al., 2022).

#### 2.3.3 Abnormal *α*-syn aggregation

*α*-syn aggregation, a characteristic of PD pathophysiology, might be impacted by gut dysbiosis. Altered microbiota promotes *α*-syn misfolding in the ENS and gastrointestinal epithelial cells, promoting pro-inflammatory immune activation and facilitating its spread to the CNS (Sterling et al., 2022). On the one hand, it has been shown that certain gut bacteria or their secreted metabolites promote the aggregation and dissemination of *α*-syn. In mice with *leucine-rich repeat kinase 2* gene, Liang et al. (2023) demonstrated that giving them *Escherichia coli* by mouth caused curli-mediated phosphorylation and accumulation of *α*-syn in the colon, which then spread along the gut-brain axis to the CNS. Additionally, hemolysin A secreted by *Proteus mirabilis* triggers *α*-syn oligomerization via activation of Mechanistic Target of Rapamycin-dependent autophagy signaling pathways in intestinal cells, ultimately inducing motor deficits and neurodegeneration (Huh et al., 2023). Notably, *Dubosiella* has been implicated in *α*-syn aggregation via the suppression of branched-chain amino acid catabolism, leading to the peripheral accumulation of valine and isoleucine, which disrupts lysosomal function and hinders *α*-syn clearance (Wu et al., 2025). On the other hand, Wang et al. (2022) discovered that the probiotic *Lactobacillus plantarum* DP189 could suppress oxidative stress, restore microbial diversity, and decrease *α*-syn aggregation in the SN of PD mice, thus delaying disease progression. The findings indicate that targeted modulation of gut microbiota could be a possible therapeutic approach to reduce *α*-syn aggregation in PD pathogenesis.

#### 2.3.4 Increase in oxidative stress

Dysbiosis can worsen oxidative stress by changing microbial metabolism and decreasing antioxidant metabolite production. This promotes neuronal damage and *α*-syn misfolding in the ENS, which subsequently spreads to the CNS (Bullich et al., 2019; Shandilya et al., 2022). Studies have shown that *Akkermansia* increases intestinal permeability, exposing neurons to oxidative conditions that favor *α*-syn aggregation (Nishiwaki et al., 2020b). Yu et al. (2023) found that gut dysbiosis aggravated oxidative stress responses and neurobehavioral impairments by downregulating *Nicotinamide mononucleotide adenylyltransferase 2*, a gene involved in NAD^+^ synthesis, in PD rat models. Emerging evidence indicates that the modulation of gut microbiota can reduce oxidative stress responses. Zhu et al. (2025) demonstrated that sleep deprivation promotes the synthesis of microbiota-derived adenosine, which elevates the production of reactive oxygen species by upregulating the pro-oxidant enzyme NADPH oxidase 4 and inhibiting the antioxidant factor Nuclear factor erythroid 2-related factor 2, consequently exacerbating oxidative damage to dopaminergic neurons. Probiotic supplements significantly mitigated these effects. Nurrahma et al. (2022) revealed that a high dose of the mangosteen pericarp, abundant in antioxidants, restores gut microbiota balance by diminishing pro-inflammatory bacterial genera (e.g., *Sutterella*, *Rothia*, *Aggregatibacter*), which exhibited a negative correlation with antioxidant gene expression. This improves antioxidant levels and alleviates PD motor deficits. Additionally, Gao et al. (2024) demonstrated that in 1-methyl-4-phenyl-1,2,3,6-tetrahydropyridine (MPTP)-induced PD mice, administration of ginkgolide C could restore gut microbiota homeostasis, exert antioxidant effects by activating the Protein Kinase B/Nuclear factor erythroid 2-related factor 2/Heme oxygenase-1 pathway in SN4741 neuronal cells, and alleviate pathological damage in mice.

#### 2.3.5 Reduction in production of neurotransmitters

It has been shown that the gut microbiota synthesize numerous neurotransmitters that are present in the human brain, such as DA, serotonin, *γ*-aminobutyric acid, and noradrenaline. Gut microbiota dysbiosis may disrupt neurotransmitters synthesis, perturbing the CNS homeostasis through gut-brain axis signaling pathways, which may contribute to the pathological progression of PD neurological dysfunction (Strandwitz, 2018; Wang et al., 2021). Research by Gao et al. (2018) revealed that when gut microbiota in experimental animals was changed through antibiotic infusion, there were significant reductions in the concentrations of serotonin, DA, and aromatic amino acids in their blood and hypothalamus compared to a control group infused with normal saline. This discovery emphasizes how vital the gut bacteria is to preserving neurotransmitter and precursor levels. Similarly, van Kessel et al. (2019) investigated the impact of microbial tyrosine decarboxylase in the proximal small intestine—a primary area for Levodopa absorption—in PD patients. They observed an increase in tyrosine decarboxylase activity, which led to premature transformation of L-dopa, significantly reducing its plasma levels and bioavailability. This, in turn, increased therapeutic dose requirements and reduced drug efficacy. According to these findings, gut dysbiosis may directly or indirectly influence the pharmacokinetics, bioavailability, and side effects of medications used to treat PD.

Overall, the findings presented underscore the role that gut microbiota plays in regulating neurotransmitter production and its profound implications for PD pathophysiology as well as the optimization of therapy approaches.

### 2.4 Metabolites of gut microbiota

The metabolites produced by the gut microbiota play a critical role in regulating neurodegenerative diseases, such as PD, via the MGBA. The pathological mechanisms mediated by four key metabolite classes are outlined.

#### 2.4.1 SCFAs

SCFAs, including acetate, propionate, and butyrate, are microbial metabolites derived from anaerobic fermentation of dietary fibers. Emerging evidence establishes a link between SCFA homeostasis disruption and neurodegenerative pathogenesis. In PD, reduced fecal SCFA levels compromise the structural integrity of the intestinal barrier and the blood–brain barrier (BBB), promote *α*-syn pathological aggregation, and exacerbate intestinal inflammation and neuroinflammation (Chen et al., 2022; Duan et al., 2023). Preclinical studies demonstrate that SCFA supplementation attenuates dopaminergic neurodegeneration and alleviates motor impairments in PD mice by inhibiting NF-κB/mitogen-activated protein kinase pathway inhibition in the SN and reducing *α*-syn aggregation (Hou Y. F. et al., 2021; Hou Y. et al., 2021). The neuroprotective effects of SCFAs extend to AD pathophysiology by modulating synaptic plasticity, amyloid-*β* (Aβ) and tau pathology, and neuroinflammation (Tang et al., 2022). Clinically, mild cognitive impairment patients exhibit a significant reduction of fecal SCFAs that inversely correlates with Aβ burden in cognition-associated brain regions (Gao et al., 2023). Notably, in models of amyotrophic lateral sclerosis, the abundance of butyrate-producing bacteria decreases in *SOD1*^G93A^ mice, whereas butyrate supplementation enhances gut barrier integrity, reduces *SOD1*^G93A^ aggregates, decelerates motor neuron degeneration, and prolongs survival of these mice (Loh et al., 2024).

#### 2.4.2 Secondary bile acids

Intestinal microbiota mediate the biotransformation of primary to secondary bile acids. In PD patients, increased levels of deoxycholic acid and lithocholic acid in the cecum are closely related to increased abundance of bile acid-synthesizing microbiota. These secondary bile acids induce pathologic *α*-syn aggregation and propagation through exerting pro-inflammatory and cytotoxic effects while simultaneously impairing mitochondrial function and autophagy regulation, contributing to neurodegenerative disease pathology (Castro-Caldas et al., 2012; Kiriyama and Nochi, 2023). Notably, taurodeoxycholic acid, a neuroprotective bile acid, demonstrates therapeutic potential across neurodegenerative models. In PD mice, taurodeoxycholic acid administration significantly delays dopaminergic neurodegeneration by inhibiting the c-Jun N-terminal kinase apoptosis pathway, reducing mitochondrial reactive oxygen species, and activating the Protein Kinase B survival pathway (Li et al., 2021). AD rodent models further reveal the capacity of taurodeoxycholic acid to reduce Aβ deposition in the hippocampus and prefrontal cortex and rescue cognitive deficits in spatial, recognition, and contextual memory domains (Lo et al., 2013).

#### 2.4.3 Trimethylamine N-oxide

The gut microbiota mediates enzymatic conversion of dietary choline and carnitine to trimethylamine, which undergoes hepatic oxidation to generate TMAO, a compound implicated in neurodegeneration through various mechanisms (Caradonna et al., 2025). Clinical metabolomic profiling reveals elevated circulating TMAO concentrations in PD patients, though independent of disease progression (Voigt et al., 2022). Mechanistically, TMAO promotes abnormal *α*-syn conformational changes and pathological aggregation and activates pro-inflammatory signaling pathways, such as NF-κB. Additionally, TMAO penetrates the BBB, exacerbating neuroinflammation and neuronal damage (Caradonna et al., 2024). Lee et al. (2022) demonstrated that TMAO-treated midbrain organoids showed impaired brain-derived neurotrophic factor signaling, loss of dopaminergic neurons, astrocyte activation, and neuromelanin accumulation. Furthermore, TMAO induced the pathological phosphorylation of *α*-syn and tau proteins, facilitating their aggregation. Vogt et al. (2018) identified a correlation between elevated TMAO levels and AD pathology and markers of neuronal degeneration in the cerebrospinal fluid. Individuals with mild cognitive impairment and AD dementia exhibited higher TMAO levels in the cerebrospinal fluid compared to cognitively normal individuals.

#### 2.4.4 Lipopolysaccharide

LPS, an endotoxin produced by Gram-negative bacteria, plays a multifaceted role in neurodegenerative pathology. Gorecki et al. (2019) found that PD patients had a significantly higher abundance of LPS-producing Gammaproteobacteria in the gut compared to healthy controls, and LPS reduces the expression and disrupts the distribution of intestinal epithelial tight junction markers (e.g., Zonula Occludens-1, e-Cadherin). A clinical study indicated that plasma LPS rose with cognitive decline, and in non-dementia participants, high plasma LPS was independently linked to mild cognitive impairment (Saji et al., 2022). LPS activates TLR4 receptors, triggering downstream Myeloid differentiation primary response protein 88 and TIR-domain-containing adapter-inducing interferon-*β* pathways. It also induces the release of pro-inflammatory cytokines (e.g., TNF-α, Interleukin-1 beta) from microglia and astrocytes, causes oxidative stress and mitochondrial dysfunction, promotes Aβ deposition, Tau hyperphosphorylation, and *α*-syn aggregation, and results in neuronal and synaptic damage, thus driving neurodegeneration (Batista et al., 2019; Kesika et al., 2021; Brown et al., 2023).

In summary, the metabolites of gut microbiota regulate organismal homeostasis through complex mechanisms, and their dysregulation may raise the risk of neurodegenerative diseases. Thus, targeting the generation or signaling pathways of these metabolites may provide potential therapeutic strategies for PD and other neurodegenerative conditions.

## 3 FMT treatment for PD

For PD, the gut microbiota has become a potential treatment focus. Restoring gut microbiota balance to delay or prevent neurodegeneration in PD represents a novel treatment strategy. Interventions that target the gut microbiota include antibiotics, probiotics, prebiotics, dietary modifications, and FMT. Among these, FMT has drawn a lot of interest as a novel and promising approach for treating PD (Varesi et al., 2022).

### 3.1 Definition and application of FMT

To restore gut microbiota diversity and function, FMT entails transplanting functional intestinal flora from healthy donors’ feces into the patients’ gastrointestinal tract. This procedure aims to increase beneficial bacteria, reduce harmful bacterial populations, and re-establish gut homeostasis, thereby mitigating disease progression. According to data from ClinicalTrials.gov, more than 400 FMT-related clinical trials have been registered worldwide, underscoring its growing prominence in medical research.

FMT can be administered through two primary methods: capsule transplantation and bacterial liquid transplantation. Bacterial liquid transplantation is further divided into three pathways: (1) the upper gastrointestinal tract route, using nasogastric or nasojejunal tubes or a gastroscope to introduce the transplant; (2) the colonoscope route, involving the insertion of a colonoscope to deliver fecal bacteria to the ileum; and (3) the enema route (König et al., 2016). Donor selection for FMT requires stringent screening criteria, including eight dimensions of assessments. Standardized effectiveness criteria for FMT are currently lacking.

Existing evidence demonstrates the short-term safety of FMT, with most adverse events being mild, self-limiting gastrointestinal symptoms such as abdominal discomfort, diarrhea, constipation, borborygmi, bloating, nausea, and vomiting. Serious adverse events are rare but warrant investigation to improve safety protocols. Notably, the US Food and Drug Administration has approved FMT for the treatment of *Clostridium difficile* infection, achieving cure rates of approximately 90% (Carlucci et al., 2016).

Beyond *Clostridium difficile* infection, FMT holds potential for a variety of diseases linked to gut microbiota dysbiosis, including ulcerative colitis, irritable bowel syndrome, sepsis, depression, type 2 diabetes, autism spectrum disorder, multiple sclerosis, PD, AD, epilepsy, Guillain-Barré syndrome, and amyotrophic lateral sclerosis (Li et al., 2019; Kim et al., 2020b; Kim et al., 2020a; Vendrik et al., 2020; Wang et al., 2020; Cui et al., 2021; Chen et al., 2023). This broad applicability underscores the promising future of FMT in both intestinal and systemic diseases linked to microbiota dysregulation.

### 3.2 FMT and PD

FMT has been investigated in preclinical and clinical research for PD. As these studies consistently demonstrate, FMT can effectively restore gut microbiota dysbiosis associated with PD (Table 2).

**Table 2:** The application of FMT for PD: preclinical and clinical research.

Ref.	Models	Fece donors	Fece recipients	Microbiota changes	
				↑	↓
Zhao et al. (2021)	mice	the control group mice	Rotenone-induced PD mice	Proteobacteria, Helicobacteraceae, Lactobacillaceae, Enterobacteriaceae, *Barnesiella*, *Roseburia*, *Butyricicoccus*, *Helicobacter*	Verrucomicrobia, Coriobacteriaceae, *Akkermansia*, *Desulfovibrio*
Sun et al. (2018)	mice	normal control mice/PD mice	MPTP+FMT group, NS + PD-FMT group, NS + FMT group	Firmicutes, Clostridiales	Proteobacteria, Turicibacterales, Enterobacteriales
Xie et al. (2023)	mice	PD patients/ healthy human controls	MPTP+PD FMT group, MPTP+HC FMT group	Verrucomicrobiota, *Akkermansia*	Unclassified Muribaculaceae, *Odoribacter*
Kuai et al. (2021)	human	The China fmtBank (Nanjing, China)	11 PD patients with constipation	*Blautia*, *Prevotella*	Bacteroidetes
Xue et al. (2019)	human	a healthy 20-year-old female	a male PD patient who refused to take drugs because of hallucinations	*Ruminococcus*, *Blautia*, Prevotellaceae, *Faecalibacterium*	*Bacteroides*
DuPont et al. (2023)	human	4 thoroughly screened donors	8 PD patients with constipation	Firmicutes	Proteobacteria
Huang et al. (2019)	human	a 26-year-old male	a 71-year-old male patient presented with intractable constipation	Firmicutes	Proteobacteria, Bacteroidetes

#### 3.2.1 Preclinical studies

**Figure 2:**
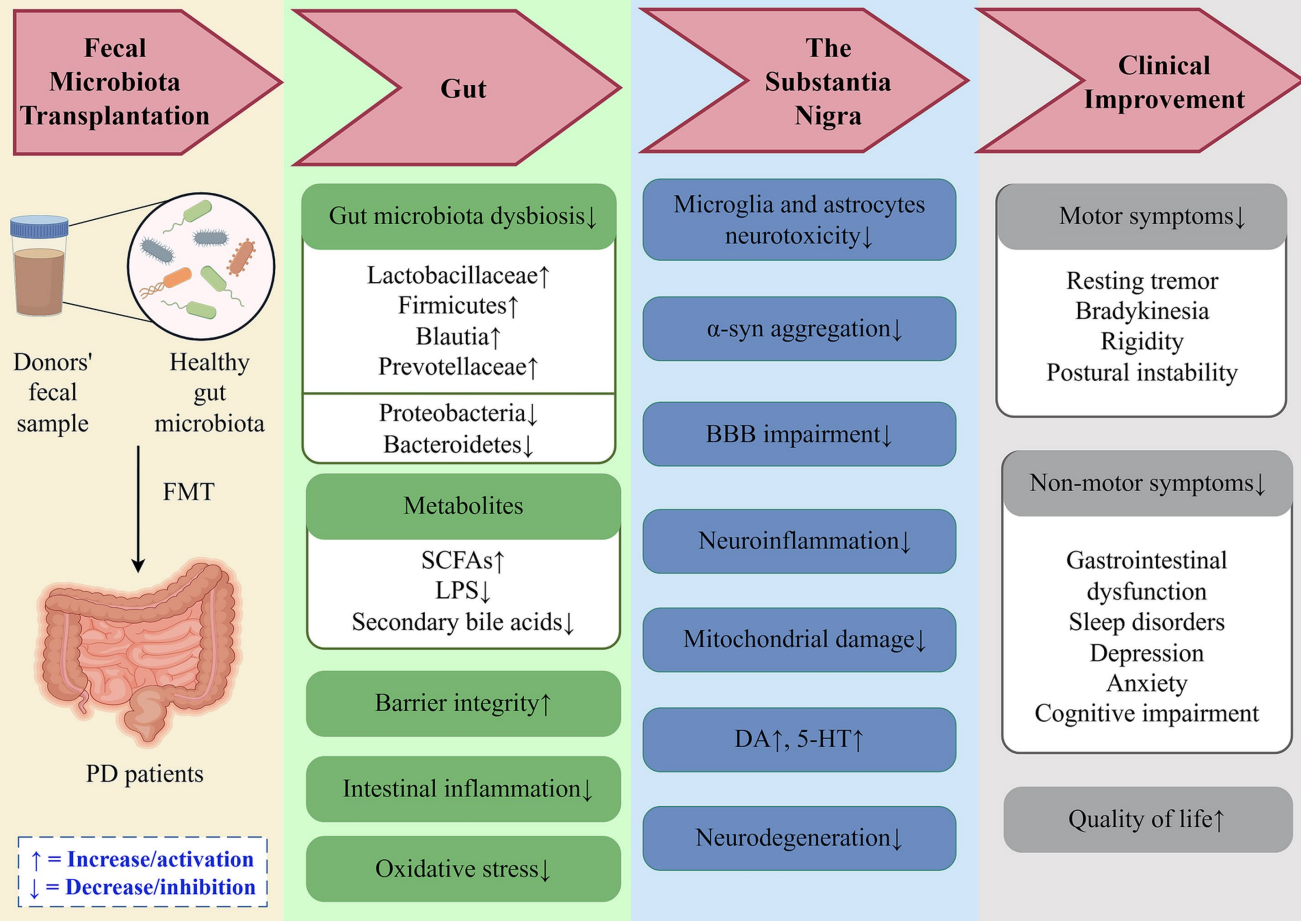
Potential mechanisms of fecal microbiota transplantation in PD treatment. FMT restores gut microbial homeostasis in PD patients by correcting dysbiosis, enhancing intestinal barrier integrity, and reducing oxidative stress and inflammatory responses. These effects mitigate neurodegeneration through modulation of the gut-brain axis, ultimately improving clinical manifestations and quality of life. DA, Dopamine; 5-HT, 5-Hydroxytryptamine; *α*-syn, *α*-synuclein; BBB, Blood–Brain Barrier; SCFAs, Short-Chain Fatty Acids; LPS, Lipopolysaccharide.

Preclinical evidence reveals several key mechanisms through which FMT improves gastrointestinal function, alleviates motor symptoms, and delays neurodegeneration in PD (Figure 2):

1. **Reduction of Inflammatory Effects and Oxidative Stress**: FMT relieves the neurotoxic effects of microglia and astrocytes, lowers LPS in the colon and SN, and reduces the secretion of pro-inflammatory cytokines while elevating anti-inflammatory factors. Moreover, FMT modulates inflammatory signaling pathways, including TLR4/TANK-binding kinase 1/NF-κB/TNF-*α* (Sun et al., 2018), TLR4/Phosphatidylinositol 3-kinase/Protein Kinase B/NF-κB (Zhong et al., 2021), and TLR4/Myeloid differentiation primary response protein 88/NF-κB (Zhao et al., 2021). In addition, Xie et al. (2023) confirmed that FMT activates the AMP-activated protein kinase/Superoxide dismutase 2 pathway, mitigating mitochondrial damage and enhancing mitochondrial antioxidative capacity. Studies have indicated that FMT reduced oxidative stress induced by 6-Hydroxydopamine in PD rat models, a known contributor to PD progression (Yu et al., 2023).
2. **Reduction of ***α***-syn Aggregation**: Transplantation of fecal microbiota from PD patients into mice has been shown to promote microglial activation and *α*-syn aggregation by modulating metabolites such as SCFAs, which exacerbates motor dysfunction (Sampson et al., 2016). In PD mouse models, FMT has been reported to restore gut microbiota diversity, elevate SCFA levels (especially butyrate), and reduce pathological *α*-syn aggregation in both the ENS and SN, ultimately ameliorating motor dysfunction (Sun et al., 2018; Liang et al., 2023). Ni et al. (2025) demonstrated that FMT may regulate SCFA levels by upregulating SCFA receptors Free Fatty Acid Receptor 2 and Free Fatty Acid Receptor 3, thereby mitigating pathological features. Fang et al. (2024) found that rotenone-induced gut dysbiosis promotes *α*-syn transcription via activation of the CCAAT/Enhancer-Binding Protein Beta/Asparagine Endopeptidase pathway, while FMT alleviates this pathological damage.
3. **Restoration of BBB Integrity**: FMT has been demonstrated to enhance BBB integrity and mitigate dopaminergic neuronal damage, thereby exerting neuroprotective effects. Studies have revealed that compared to normal controls, germ-free mice and antibiotic-treated mice with gut microbiota depletion exhibit significantly increased BBB permeability. FMT can upregulate the expression of tight junction proteins in the CNS, including Zonula Occludens-1, Zonula Occludens-2, occludin, and claudin-5, thereby restoring BBB integrity and reducing its permeability (Braniste et al., 2014; Sun N. et al., 2021). In PD mouse models, Zhao et al. (2021) found that FMT treatment ameliorated the tight junction structure defects in the SN, alleviated endothelial cell damage, and significantly upregulated the Messenger RNA levels of tight junction proteins.

#### 3.2.2 Clinical investigations

Clinical trials involving FMT in PD patients further support these preclinical findings. Fecal samples collected before and after FMT have undergone microbiota sequencing, revealing significant restoration of gut microbiota composition. Symptom assessments using scales such as the Unified Parkinson’s Disease Rating Scale, Non-Motor Symptoms Scale, and Parkinson’s Disease Questionnaire-39 indicate improvements in motor symptoms, constipation, anxiety, depression, sleep, and cognitive function, all of which improve overall quality of life. Furthermore, adverse events are less common and generally self-limiting (Xue et al., 2019; Kuai et al., 2021; Segal et al., 2021; Cheng et al., 2023; DuPont et al., 2023; Liu et al., 2023).

However, FMT’s therapeutic effects appear time-dependent. Research indicates that microbiota composition and related motor and non-motor symptoms, except constipation, may partially revert after a certain period post-transplantation (Huang et al., 2019; Xue et al., 2019). Further investigation is needed to determine long-term efficacy and stability. Additionally, transplantation methods may impact therapeutic outcomes. For instance, Xue et al. (2020) compared colonoscope-administered FMT with nasojejunal tube administration and found that the former yielded superior clinical benefits.

#### 3.2.3 Single-strain microbiota transplantation

Recent studies highlight the promising potential of single-strain microbiota transplantation in PD treatment through modulation of the MGBA. *Lactobacillus plantarum* PS128, a probiotic strain, has been shown to alleviate motor deficits in PD mice through multi-target mechanisms. Specifically, PS128 restores gut microbiota homeostasis, diminishes neuroinflammation via the microRNA-155-5p/Suppressor of Cytokine Signaling 1 pathway, inhibits the neurotoxic activation of microglia and astrocytes, alleviates oxidative stress damage, protects dopaminergic neurons, and ultimately mitigates neurodegeneration (Liao et al., 2020; Lee et al., 2023). Clinical trials further support its therapeutic efficacy, with PS128 supplementation demonstrating significant improvements in motor symptoms and quality of life in PD patients (Lu et al., 2021).

Additionally, other microbial strains have shown promise in the treatment of PD. For example, *Bifidobacterium breve* (CCFM1067, Bif11) and *Lacticaseibacillus rhamnosus* E9 demonstrate neuroprotective effects in PD mouse models by enhancing intestinal barrier integrity and alleviating pathological progression (Li T. et al., 2022; Aktas et al., 2024; Valvaikar et al., 2024). Oral administration of *Clostridium butyricum* has been shown to restore colonic Glucagon-Like Peptide-1 (GLP-1) and G Protein-Coupled Receptor 41/43 levels, along with cerebral GLP-1 Receptor expression in PD mice, thereby mediating neuroprotection via the GLP-1/GLP-1 Receptor pathway (Sun J. et al., 2021).

These findings collectively emphasize the therapeutic potential of single-strain microbiota transplantation in mitigating PD progression. Future studies should aim to elucidate the molecular mechanisms underlying single-strain interventions and validate the long-term safety and therapeutic efficacy through rigorous clinical trials.

## 4 Discussion

In summary, the bidirectional regulation and communication through the MGBA provide an innovative framework for investigating the pathological processes underlying PD. There is much evidence now available linking gut microbiota dysbiosis, its metabolites, and PD initiation and progression. While characteristic alterations in the composition have been shown in PD, inconsistencies across studies suggest that a consensus on specific microbial alterations has yet to be reached. Nevertheless, FMT has demonstrated potential in alleviating clinical symptoms and delaying PD progression.

Despite the fact that several research studies have verified the short-term effectiveness and safety of FMT, the field remains in its early stages with limited clinical trials. Most existing research comprises cross-sectional comparisons between PD patients and healthy people, with limited follow-up investigations tracking long-term outcomes. The long-term safety and sustained effectiveness thus require further exploration.

In PD, the gut microbiota represents an emerging potential therapeutic target. However, to fully assess the clinical utility, future research should focus on (1) elucidating the molecular mechanisms underlying gut dysbiosis in PD; (2) conducting rigorous, high-quality clinical trials to validate the efficacy and safety of FMT; and (3) optimizing FMT protocols by determining the optimal transplantation routes, dosing regimens, and administration frequencies. The development of standardized treatment guidelines would facilitate the responsible translation of FMT into clinical practice. Provided that ongoing research continues to demonstrate both safety and efficacy, FMT may potentially emerge as an adjunctive approach in PD management.
